# NGAL protects against endotoxin-induced renal tubular cell damage by suppressing apoptosis

**DOI:** 10.1186/s12882-018-0977-3

**Published:** 2018-07-06

**Authors:** Mei Han, Ying Li, Di Wen, Maodong Liu, Yuteng Ma, Bin Cong

**Affiliations:** 1 Department of Emergency, The Second Hospital of Hebei Medical University, Shijiazhuang, China; 2 Department of Nephropathy, The Third Hospital of Hebei Medical University, Shijiazhuang, 050051 China; 3 Department of Forensic Medicine, Hebei Medical University, Shijiazhuang, China

**Keywords:** Neutrophil gelatinase associated lipocalin, Acute kidney injury, Apoptosis, siRNA, Lipopolysaccharide

## Abstract

**Background:**

We sought to confirm that neutrophil gelatinase-associated lipocalin (NGAL) protects against apoptosis during endotoxemia.

**Methods:**

Endotoxemia was induced in rats with lipopolysaccharide (LPS; 3.5 mg/kg) and serum creatinine (SCr), urinary NGAL (uNGAL), renal histopathology confirmed acute kidney injury (AKI). Renal caspase 3 and NGAL were assayed with immunohistochemistry 6 h later. A HK-2 cell model was used in which NGAL and caspase 3 mRNA were evaluated by qRT-PCR within 6 h after LPS (50 μM) treatment, and correlations were studied. NGAL and caspase 3 mRNA expression were measured after delivering NGAL siRNA in HK-2 cells and apoptosis was measured with TUNEL and flow cytometry.

**Results:**

SCr and uNGAL were significantly increased after LPS treatment and renal morphology data indicated AKI and renal tubular epithelial cell apoptosis. Caspase 3 and NGAL were predominantly expressed in the tubular epithelial cells and there was a correlation between caspase 3 and NGAL protein (*r* = 0.663, *p* = 0.01). In vitro, there was a strong correlation between caspase 3 and NGAL mRNA in LPS-injured HK-2 cells within 24 h (*r* = 0.448, *p <* 0.05). Suppressing the *NGAL* gene in HK-2 cells increased caspase 3 mRNA 4.5-fold and apoptosis increased 1.5-fold after LPS treatment.

**Conclusions:**

NGAL is associated with caspase 3 in renal tubular cells with endotoxin-induced kidney injury, and may regulate its expression and inhibit apoptosis.

## Background

AKI occurs for more than half of ICU patients, with sepsis being the most common trigger, and the lack of sensitive and specific biomarkers to confirm renal cell injury increases mortality for septic AKI [1–3]. Recently, using genomic technology, transcriptome and proteome analysis, researchers identified NGAL as an acute phase protein (APP) and an early biological marker for AKI [4, 5]. Many studies of AKI in critically ill patients have suggested the diagnostic value of NGAL which is rapidly expressed, synthesized and secreted in body fluids by damaged kidney tissues and initial work suggests that NGAL may be a promising indicator of septic AKI [6].

Whether this APP can reduce kidney injury or reverse damage is unknown because few studies have been published to describe renal NGAL expression during AKI. Previous work suggests that [4, 7] damaged rat kidney expresses NGAL significantly in early stages of ischemic AKI, and NGAL mRNA increased 1000 times. In our early AKI rat model, renal epithelial cells after lipopolysaccharide (LPS) administration were edematous and apoptotic and NGAL mRNA expression was increased [8]. However, the function of NGAL is unclear, so we investigated renal epithelial cell death to explore the relationship between NGAL and apoptosis in a rat model and an HK-2 cell model of LPS-induced injury.

## Methods

### Experimental groups

In vivo experiments were conducted on male Sprague-Dawley rats (200 ± 20 g, 7–8 weeks old). They were purchased from the Experimental Animal Center of Hebei Medical University (Shijiazhuang, China) and maintained in a specific pathogen-free environment in our facility. Rats were fed freely with standard food and water and were cared for in accordance with the Local Committee of Animal Use and Protection of Hebei Medical University, China. Animals were housed with 12-h light-dark cycles and were acclimated for at least 1 week before experimentation. Because the response to LPS depends on temperature, the ambient temperature was set at 24 °C. Endotoxemia and AKI were induced in rats with LPS (*Escherichia coli* 0111: B4, Sigma, St. Louis, MO).

In our previous *vivo* study [8], we observed that LPS-induced the upregulation of renal NGAL mRNA from 3 to 12 h after treatment compared with controls (*p* < 0.001). At peak expression of NGAL mRNA increased 260-fold (6 h post LPS). Thus, we selected 20 rats to evaluate the relationship of NGAL and kidney injury, rats were randomized into 2 groups by medical laboratory technicians (*n* = 10 rats/group). Group 1 included controls (Con) treated with isometric sterile saline. Group 2 was an endotoxin induced AKI group (sAKI) treated with LPS (3.5 mg/kg, ip), which was used previously [8], due to its ability to induce moderate endotoxemia in rats. Because expression of NGAL mRNA peaked at 6 h in rat renal epithelia cells, we selected the 6 h time point post-LPS treatment to evaluate NGAL and kidney injury. Urine was gathered using metabolic cages (Beijing, China) and supernatant was obtained. After 6 h of LPS or saline administration, all rats were anaesthetized with sodium pentobarbital (60 mg/kg ip), and 2 mL of blood was obtained by cardiac puncture and processed to obtain serum which was frozen at − 80 °C for later analysis of SCr. Kidneys were harvested for hematoxylin and eosin (H&E) staining, transmission electron microscopy (TEM) and immunohistochemical staining (IHS). When tissue had been obtained, the animals were euthanized by dislocation of the cervical spine under deep anesthesia. Investigators were blinded to group allocation during modeling and analysis.

### Evaluating renal function

Renal function measured with serum creatinine was assessed in Con and sAKI groups using a colorimetric assay (Creatinine Assay Kit; Biosino Bio-Technology, Beijing, China) and reading changes in absorbance over 40 s in experimental samples relative to standard.

### Histological studies

Histopathology was conducted in kidney samples to determine the time-course of renal micro-morphological injury in the LPS-induced endotoxemic rats. Each kidney sample (one quarter from both the control and experimental groups) was fixed in 4% paraformaldehyde, dehydrated in graded ethanol and embedded in paraffin as previously described [9]. Each paraffin block was processed into 5-μm-thick slices and was H&E stained. A portion (~ 1 mm^3^) of renal cortex from each rat was fixed in 2.5% glutaraldehyde diluted in 0.066 M phosphate buffer (pH 7.4) for 24 h. Samples were then dehydrated in a graded ethanol series and embedded in Epon 812 resin at 60 °C for 48 h. Thin sections (50 nm) were then double-stained with uranyl acetate and lead citrate and were observed and photographed with a TEM operated at 80 kV. The epithelial layer was examined and photographed with a TEM (Hitachi, H-7500, TEM) at a magnification of 5000×.

### uNGAL measurements

Previously, urinary NGAL (uNGAL) may reflect kidney injury more than plasma NGAL (pNGAL) [8]. Thus, urine was studied with the aid of metabolic cages. uNGAL was measured using a commercially available ELISA kit (CSB-E09409r, Cusabio Biotech, Wuhan, China), according to the manufacturer’s instructions.

### Immunohistochemistry and histological scoring

NGAL and caspase 3 were evaluated by IHS. Renal tissues were fixed in 10% formalin for 24 h and subsequently embedded in paraffin. HIS was performed on 4-μM renal sections using anti-NGAL and anti-active caspase 3 (Sigma). Slides were developed using HRP-labeled secondary antibody (Dako Denmark, Shanghai, China) and DAB (Sigma). All HIS analyses were repeated at least three times and representative images are presented. Quantification of IHS was assessed in the cortex and cortico-medullary area: positive cells were counted in a high-power field (HPF, 20× magnification) or ten to fifteen high-power fields (20×) and images were obtained for each slide. Positive areas were measured using ImageJ software (NIH, Bethesda, MD) [10]. Results are shown as positive areas as a percent of the total area analyzed.

### Cell culture

HK-2 human renal proximal tubular epithelial cells were supplied from Shanghai Bioleaf Biotech (Shanghai, China). They were maintained at 37 °C in 5% CO_2_ with DMEM/F12 (Gibco Grand Island, NY) containing 10% fetal bovine serum (FBS) and penicillin (100 units/mL)-streptomycin (0.1 mg/mL) mixture (PAA, Strasse, Pasching, Austria) on plastic dishes. Cells were cultured in a humidified atmosphere at 37 °C with 5% CO_2_, and passaged twice per week. To minimize age-dependent variation, cells from passages 18–22 were used.

#### Quantitative real-time PCR (rt-qPCR)

HK-2 cells were seeded in 6-well plates at a density of 2 × 10^5^ cells per well and were exposed to LPS of 50 μM for 24 h. Cells were harvested at 1, 3, 6, 12, and 24 h after LPS-treatment, and then total RNA was extracted with TriZol Reagent (Invitrogen, Carlsbad, CA) according to the manufacturer’s instructions. RNA was measured using a Nanodrop ND-1000 spectrophotometer (Nanodrop Technologies, Wilmington, DE), and complementary DNA (cDNA) was synthesized from total RNA (500 ng) using a PrimeScriptRT regent Kit (Takara Biotechnology, Dalian, China) according to kit instructions. Subsequently, cDNA was subjected to real-time PCR using Power SYBR Green PCR Master Mix (Takara Biotechnology, Dalian, China). Each real-time PCR reaction consisted of 2 μL of diluted RT product, 10 μL SYBR Green PCR Master Mix and 250-nm specific primer pairs in a total volume of 20 μL. Reactions were performed on a 7500 real-time PCR System (Applied Biosystems, Foster, CA) for 40 cycles (95 °C for 5 s, 60 °C for 35 s) after an initial 30 s incubation at 95 °C. PCR products were separated by 2% agarose gel electrophoresis, illuminated with UV light and imaged to assess amplification. Fold changes in mRNA of each gene was calculated using the ΔΔCt method, with the housekeeping gene, GAPDH, as an internal control. NGAL, caspase 3 and GAPDH mRNA expression measured using primer sets as indicated in Table 1.

**Table 1:** The sequences of primers used for RT-PCR and NGAL siRNA

gene	sequence	product(bp)
NGAL	TTGGGACAGGGAAGACGA	240
	TCACGCTGGGCAACATTA	
Caspase3	GTTCATCCAGTCGCTTTGTGC	110
	AAATTCTGTTGCCACCTTTCG	
β-actin	TCGCGGGAGACCACCGACAC	258
	GGGGTGTTGGGTCAGGTCTCTG	
siRNA	UUUAGUUCCGAAGUCAGCUCCUUGG	
	CCAAGGAGCUGACUUCGGAACUAAA	

#### Gene silencing by siRNA

##### siRNA transfections

HK-2 cells were seeded in 6-well plates at a density of 2 × 10^5^ cells per well without antibiotics in the medium for 24 h. Orifice plates were divided into four groups: Con, LPS, siRNA and siRNA + LPS and three wells per group were used. Con and LPS-treated cells were incubated with medium, and siRNA and siRNA + LPS-treated cells were grown and transfected with 6 pM siRNA using Stealth RNAi siRNA Duplex Oligoribonucleotides and RNAiMAX ((Invitrogen, Carlsbad, CA) according to the manufacturer’s instructions. Cells were incubated for 36 h and then placed in D-MEM/F12 medium with LPS (50 μM) which was replaced in LPS and siRNA + LPS groups for 3 h and serum-free medium was used in Con and siRNA groups. To quantify NGAL and caspase 3 mRNA expression, RNA was harvested and cDNA was synthesized.

### Apoptotic assessment

HK-2 cells were seeded, transfected and treated with LPS in 6-well plates as previously mentioned, but LPS treatment was 6 h to observe apoptosis which was measured using an annexin V-FITC/ PI staining kit and flow cytometry (Becton Dickinson, San Jose, CA). After incubation, cells were washed twice with PBS and cell density was set at 1 × 10^6^/mL with precooling Hank’s Balanced Salt Solution (HBSS). Then cells were incubated with fluorescein-conjugated annexin V and PI in the dark for 15 min at room temperature. Stained cells (1 × 10^5^ cells/sample) were analyzed by flow cytometry and apoptosis was quantified.

### Measurement of apoptosis using TUNEL

The TUNEL procedure was applied to kidney sections to detect DNA fragmentation as an index of apoptosis. Counterstaining was performed with DAPI dye (1 μg/mL, Kirkegaard Perry Laboratories, Tokyo, Japan). Paraffin sections of 3 μm thick fixed with 4% paraformaldehyde in PBS were stained with a TMR red in situ Cell Death Detection Kit (Roche, Basel, Switzerland). Deparaffinized HK-2 cells were seeded at 1 × 10^5^/mL on a glass coverslip which was placed in advance in 6-well plates, transfected and treated with LPS (50 μM) as previously described, and LPS was applied for 6 h. Medium was removed and cells were fixed with 4% paraformaldehyde for 30 min at room temperature, and then cleaned with PBS twice. Next, cells were incubated with 3% H_2_O_2_ methanol solution at room temperature for 30 min, and cleaned with PBS twice. HK-2 cells were incubated in the permeabilization solution (0.1% TritonX-100 in 0.1% sodium citrate) for 2 min on ice. Cells were incubated with TUNEL reaction mixture for 60 min at 37 °C in the dark and sections were exposed to DAPI dye for 4 min in the dark. Finally, sections were mounted with VectaShield (Vector Laboratories, Orton Southgate, UK). TUNEL-positive cells were counted in 5 randomly selected fields (400× magnification) and percents were calculated against total DAPI-stained cells. Two independent observers blinded to experimental conditions performed counts and calculated average TUNEL-positive cells. Data were collected from more than 3 independent experiments performed in triplicate.

### Statistical analysis

All experiments were performed in duplicate and repeated at least three times. Data are expressed as means ± SEM. Group comparisons were performed using ANOVA (SPSS, v. 16.0, Chicago, IL). All groups were analyzed simultaneously with an LSD *t*-test and *p* < 0.05 was considered statistically significant.

## Results

**Fig. 1:**
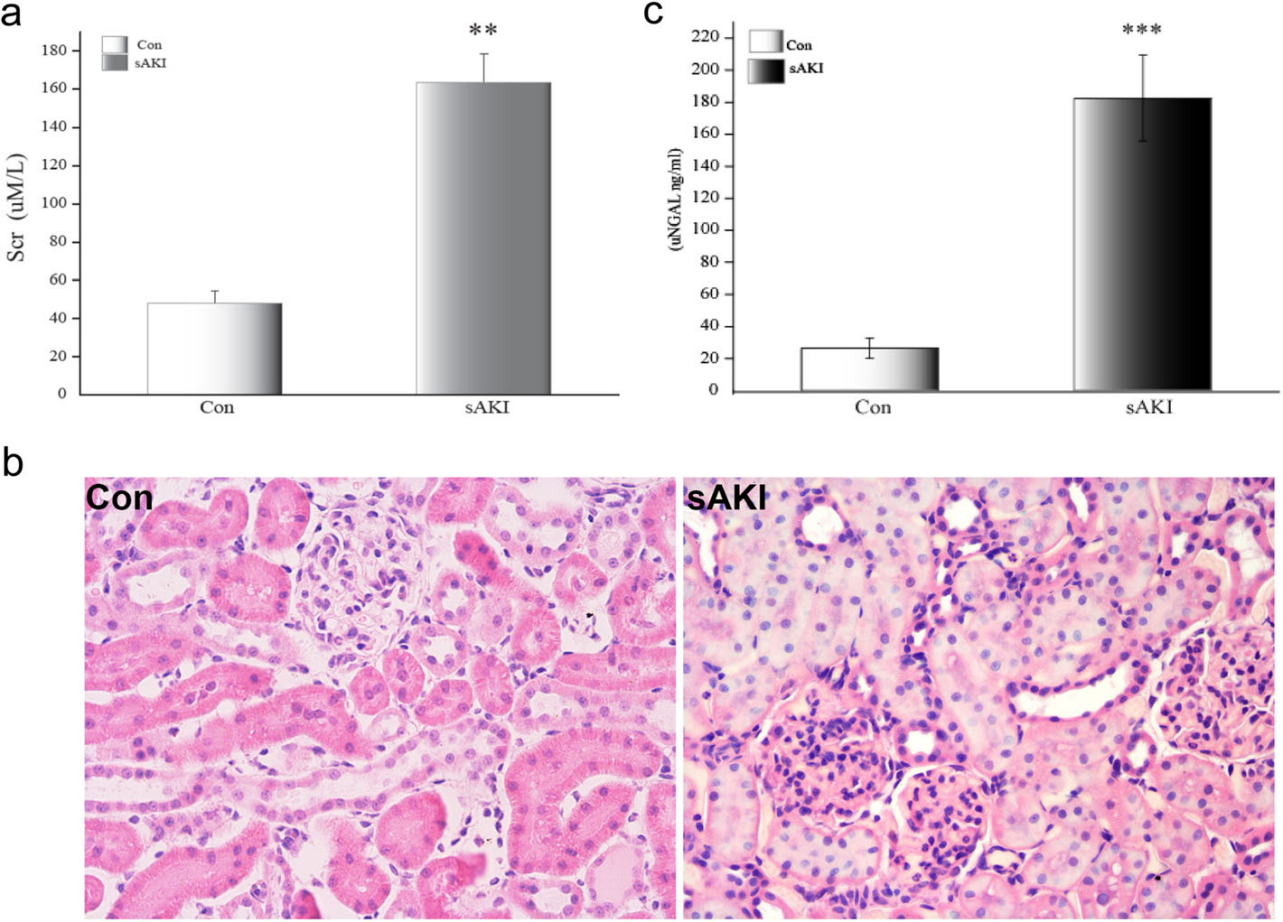
**a** SCr in rats subjected to LPS at 6 h. The data show SCr of Con and sAKI, with means ± SD values/group obtained by colorimetric assay (μM/L). SCr increased in rats with sAKI. SCr: serum creatinine, LPS: lipopolysaccharide. **p < 0.01, relative to the Con group. **b** Renal histological injury observed under light microscopy at 6 h after LPS treatment (H&E, 200×). Histopathlogical changes included renal tubular epithelial swelling and inflammatory cell infiltration without glomerular injury. **c** uNGAL in rats subjected to LPS at 6 h. Data show respective uNGAL of Con and sAKI, with means ± SD values/group obtained by ELISA (ng/mL). uNGAL was significantly increased in rats with sAKI. uNGAL: urine NGAL, LPS: lipopolysaccharide. ***p < 0.001, relative to the con group

After 6 h of LPS injection, blood was collected to measure SCr which increased almost 3.4-fold in the sAKI group compared to Con group (Fig. 1a). Renal morphology at the same time point indicated damage featuring severe tubular cell edema, cellular infiltrate and hyperemia in LPS-treated rats (Fig. 1b). Based on changes in SCr and renal histology, we confirmed that we established an LPS induced acute kidney injury animal model.

Urine sample was analyzed with ELISA. Data indicate that 6 h after LPS-treatment, uNGAL of sAKI group significantly increased, indicating pathological renal lesions in the early stage of acute rat endotoxemia (Fig. 1c).

**Fig. 2:**
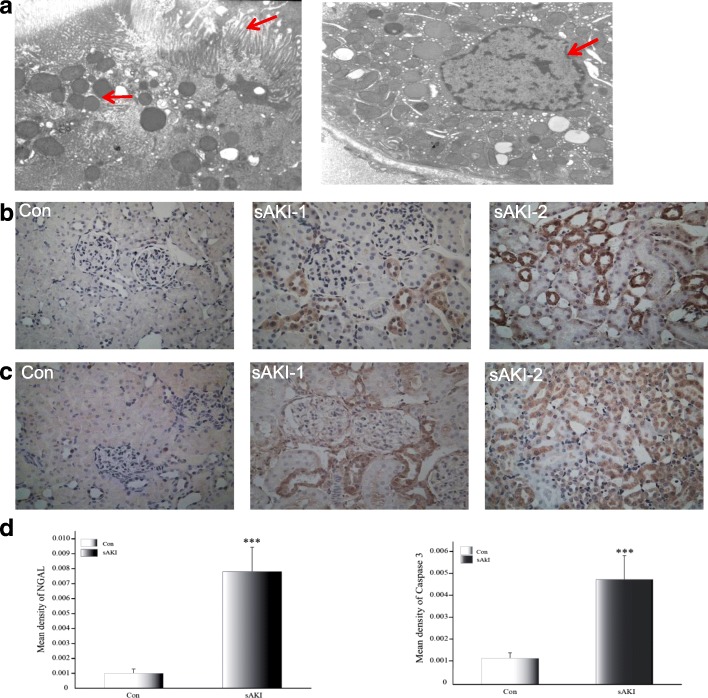
**a** Renal tubular epithelial cell injury and apoptosis observed under TEM at 6 h after LPS treatment (TEM, 5000×). Arrows indicate disarrayed microvilli, mitochondrial ballooning, and unevenly distributed nuclear chromatin in the outer nuclear layer gathered toward the center. TEM: transmission electron microscopy, LPS: lipopolysaccharide. **b** Localization of NGAL protein expression in rat kidneys under light microscopy 6 h after LPS treatment. Con: Control group, sAKI-1: renal cortex of sAKI group, sAKI-2: renal medulla of the sAKI group. (original magnification 400×). NGAL: neutrophil gelatinase-associated lipocalin, LPS: lipopolysaccharide. **c** Localization of caspase 3 protein expression in rat kidneys under light microscopy at 6 h after LPS treatment. Con: Control group, sAKI-1: renal cortex of sAKI group, sAKI-2: renal medulla of the sAKI group. Original magnification 400×. LPS: lipopolysaccharide. **d** Semi-quantification of immunohistochemical staining for NGAL and caspase 3 in kidneys of Con and sAKI rats. Data are expressed as means ± SD. ***p < 0.001, relative to controls

To confirm that LPS induced renal tubular epithelial apoptosis, proximal tubular epithelial cells of renal cortices were observed under TEM 6 h after LPS injection. Damage was evident and nuclei were apoptotic. Microvilli were visibly disordered and deficient, and we observed intracellular edema, impaired mitochondrial outer membranes, nuclear membrane contraction, and chromatin at the edge of apoptotic cells (Fig. 2a).

IHS in the sAKI group revealed that caspase 3 protein was predominantly expressed in the renal tubular epithelium and NGAL staining increased as well. No abnormal findings were observed in controls (Fig. 2b and c). Consistently, semi-quantitative analysis results showed significant differences between both groups (*p* < 0.001, Fig. 2d). IHS suggested a correlation between caspase 3 and NGAL proteins, and NGAL may increase or decrease epithelial apoptosis (*r* = 0.663, *p* = 0.01).

**Fig. 3:**
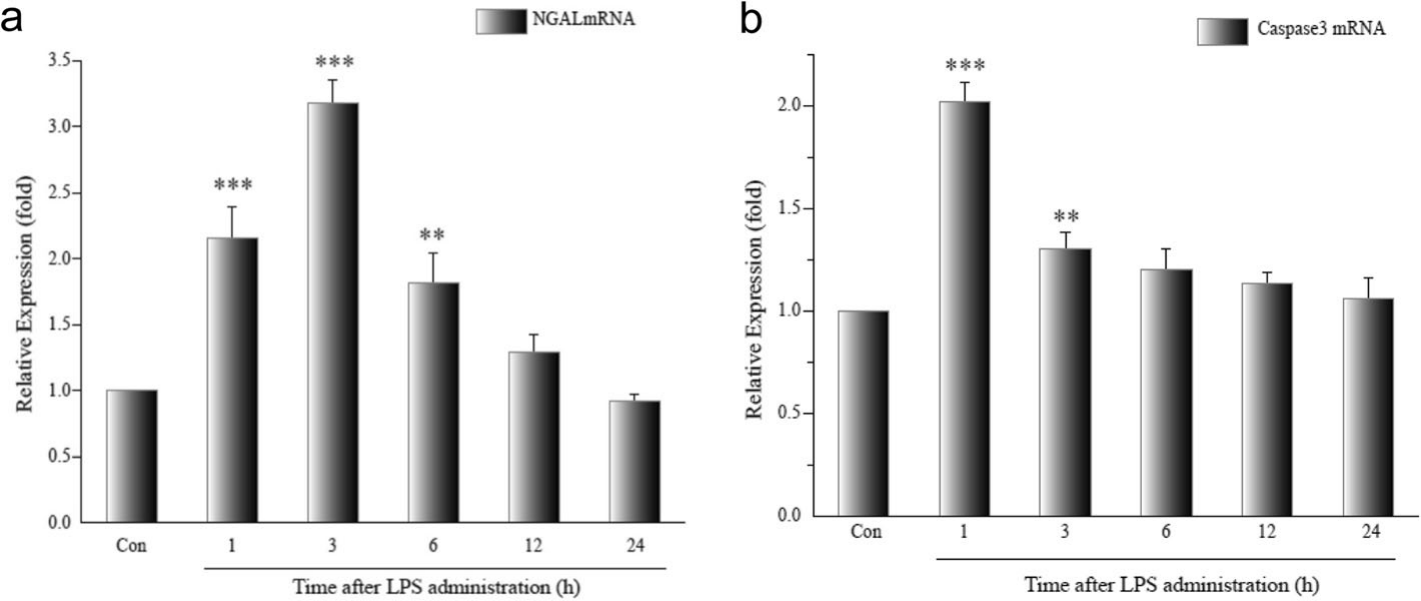
**a** Expression of NGAL mRNA in the LPS-treated HK-2. Data are means ± SD of three separate experiments in duplicate. There was a 2.2-fold increase in NGAL mRNA expression 1 h after LPS treatment, which increased to 3.2-fold at 3 h and then decreased to 1.8-fold at 6 h. NGAL: neutrophil gelatinase-associated lipocalin, LPS: lipopolysaccharide. **p < 0.01, ***p < 0.001, relative to the control group. **b** Expression of caspase 3 mRNA in the LPS-treated HK-2. Data are means ± SD of three separate experiments in duplicate. There was a 2.02-fold increase in NGAL mRNA expression at 1 h after LPS treatment, and this decreased to 1.3-fold at 3 h. **p < 0.01, ***p < 0.001, relative to controls. NGAL: neutrophil gelatinase-associated lipocalin, LPS: lipopolysaccharide

NGAL mRNA and caspase 3 mRNA were evaluated by qRT-PCR in HK-2 cells after LPS (50 μM) treatment. NGAL mRNA was significantly increased within 6 h compared with controls (LPS 1 and 3 h groups, *p <* 0.001; LPS 6 h group, *p <* 0.01), and decreased to baseline after 12 h (*p >* 0.05). Peak expression of NGAL occurred 3 h after LPS treatment (Fig. 3a). caspase 3 mRNA expression was upregulated within 3 h after LPS administration (LPS 1 h group, *p <* 0.001; LPS 3 h group, *p <* 0.05) and decreased to baseline after 6 h. Expression after 6 and 12 h was not different than controls (LPS 6 and 12 h groups, *p >* 0.05). Peak expression of caspase 3 mRNA occurred in the LPS 1 h group and was almost twice greater than controls (Fig. 3b). caspase 3 and NGAL mRNA were correlated (*r* = 0.448, *p <* 0.05).

**Fig. 4:**
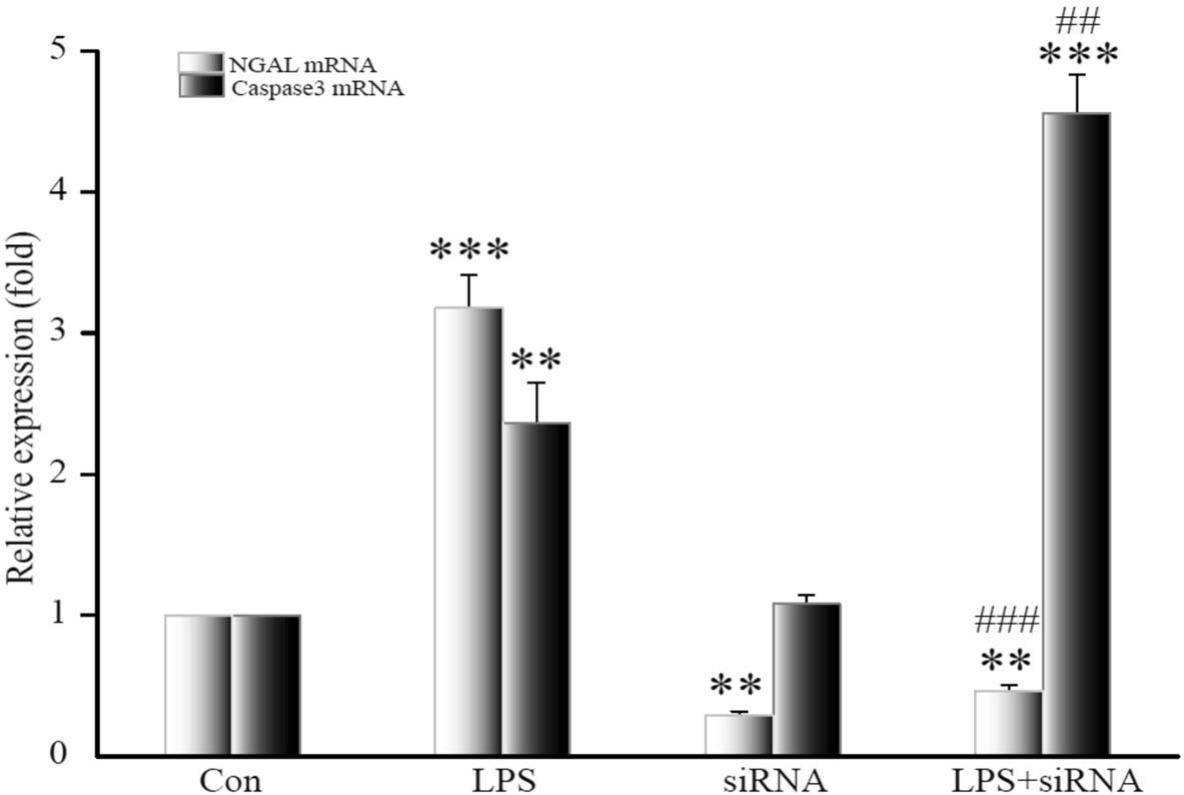
Expression of NGAL mRNA and caspase 3 mRNA in HK-2 after NGAL siRNA transfection. Data are means ± SD of three separate experiments in duplicate. **p < 0.01, ***p < 0.001, relative to controls. ^##^*p* < 0.01, ^###^*p* < 0.001, relative to the LPS group. NGAL: neutrophil gelatinase-associated lipocalin

We measured NGAL mRNA and caspase 3 mRNA expression after NGAL siRNA transfection after LPS stimulation of HK-2 cells. NGAL mRNA expression was significantly increased in LPS cells (*p <* 0.001), and suppressed in siRNA-treated cells compared to controls (*p <* 0.01). NGAL mRNA expression of siRNA + LPS-treated cells was also significantly suppressed compared to controls (*p <* 0.01) and less than 20% when compared to LPS cells (*p <* 0.001), but slightly higher than in siRNA cells (*p >* 0.05). Caspase 3 mRNA also increased significantly in LPS-treated cells (*p <* 0.01) and was not different than controls in siRNA-treated cells (*p >* 0.05). Caspase 3 mRNA of siRNA + LPS-treated cells increased compared to controls (*p <* 0.001) and was twice that of LPS-treated cells (*p <* 0.01; Fig. 4).

**Fig. 5:**
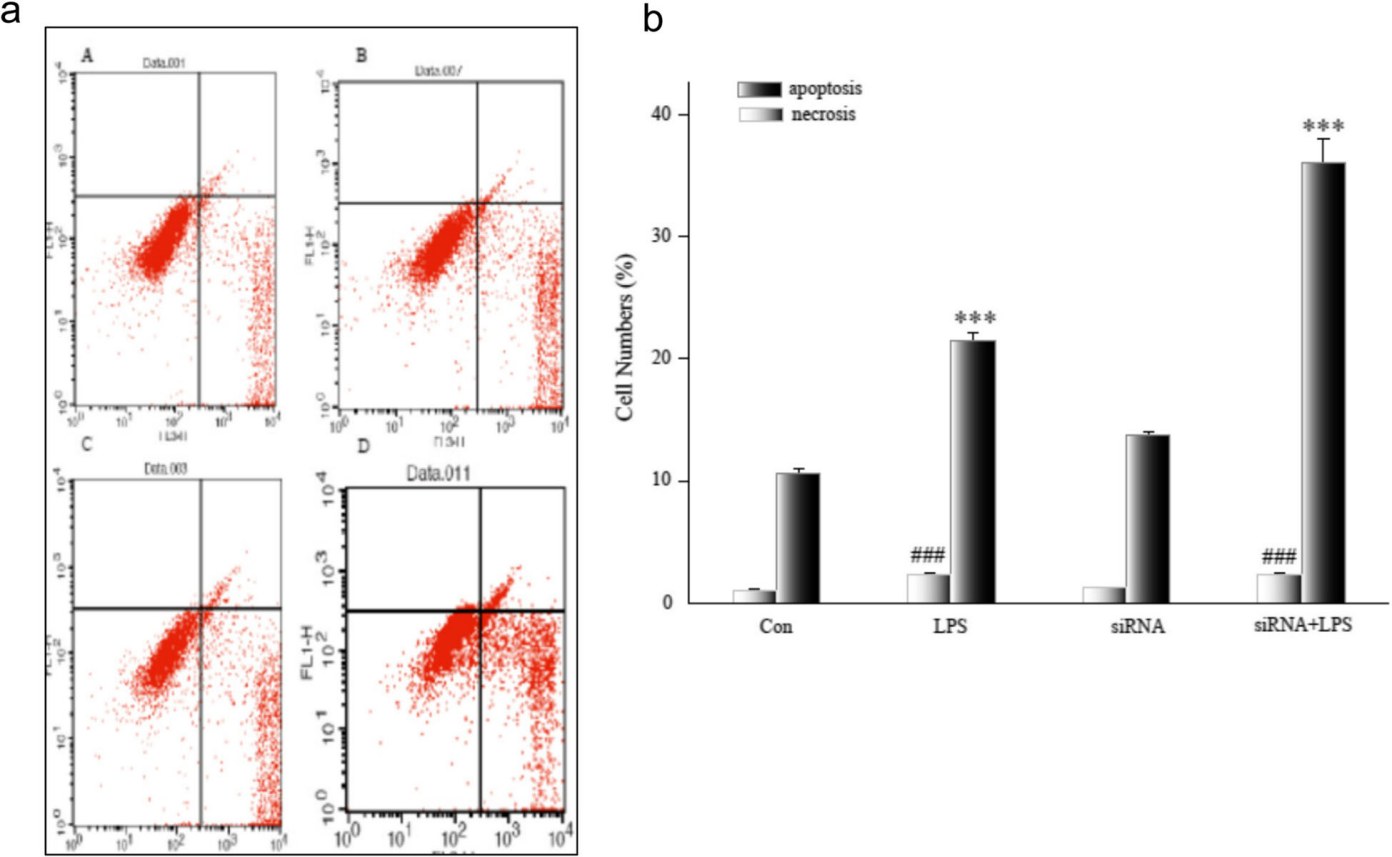
**a** Apoptosis and necrosis of HK-2 after NGAL siRNA3 transfection. Flow cytometry data for HK-2 after transfection. Necrotic cells appear in upper right quadrant, and apoptotic cells appear in lower right quadrant. A, Con group; B, LPS group; C, siRNA group; D, siRNA+ LPS group. NGAL: neutrophil gelatinase-associated lipocalin, LPS: lipopolysaccharide. **b** Apoptosis and necrosis of HK-2 after NGAL siRNA3 transfection. Flow cytometry data for four groups. Data are means ± SD of three separate experiments in duplicate. NGAL: neutrophil gelatinase-associated lipocalin, ***p < 0.001, relative to apoptosis controls. ^###^*p* < 0.001, relative to necrosis controls

**Fig. 6:**
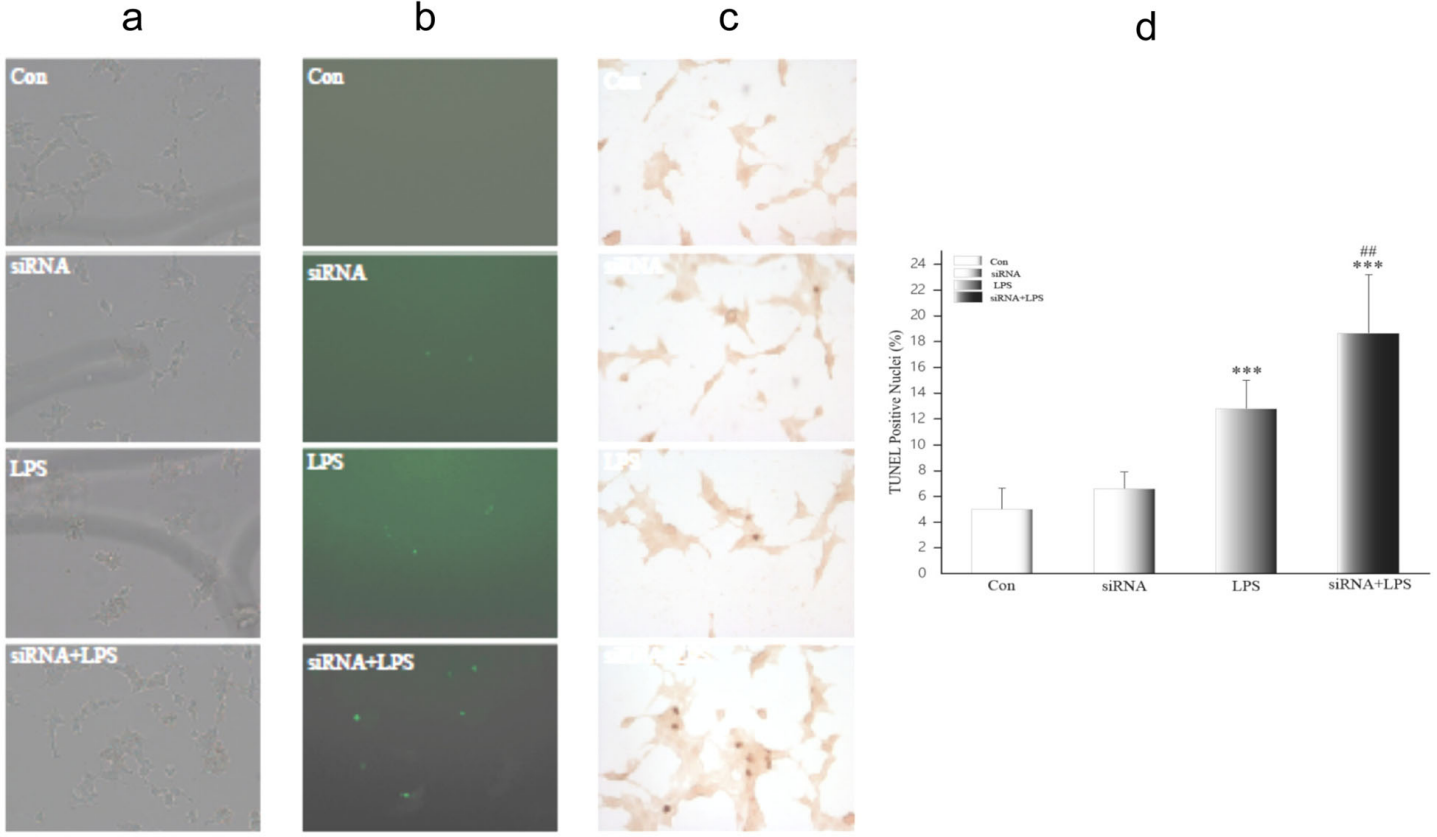
Apoptotic cells in HK-2 after transfection observed by TUNEL. **a** HK-2 observed under light microscopy (200×). **b** HK-2 observed under a fluorescent microscope, apoptotic cells are fluorescent green (200×). **c** TUNEL-positive cells observed under light microscopy, and apoptotic cells are dark brown (200×). **d** TUNEL-positive cells counted and expressed as means ± SD. TUNEL: terminal-deoxynucleoitidyl transferase mediated nick end labeling. ***p < 0.001, relative to the control group. ^##^*p* < 0.01, relative to the LPS group

Flow cytometry measurement of necrosis and apoptosis were assayed and increased only slightly after siRNA transfection and did not differ from controls (*p >* 0.05). NGAL siRNA did not injure HK-2 cells. But necrosis and apoptosis in HK-2 cells after LPS treatment were significantly greater than in controls (*p <* 0.001). In siRNA + LPS-treated cells, necrosis was significantly greater compared to controls (*p* < 0.001), but there was no difference compared to LPS-treated cells (*p >* 0.05). Apoptosis in siRNA + LPS-treated cells was significantly greater than in the other three groups (Fig. 5a and b). Furthermore, the effect of NGAL siRNA on tubular cell apoptosis according to TUNEL staining showed similar results. There were few TUNEL-positive cells in sham HK-2 cells with/without transfection, but many TUNEL-positive cells appeared in LPS-treated cells. siRNA + LPS-treated cells were the most apoptotic (Fig. 6). Thus, inhibition of NGAL aggravated endotoxin-induced renal tubular cell damage.

## Discussion

NGAL and matrix metalloproteinase-9 (MMP-9) was observed during a study of gelatinase (92 kDa) in neutrophil granulocytes in 1993 by Kjeklsen’s group [3, 11]. Then the complete genome sequence of NGAL cDNA was cloned and identified in 1994 and 1997, respectively [12, 13]. Recently, using gene expression microarray Mishra and colleagues reported that *NGAL* gene expression in the kidney increased in early stage AKI in animal models. Down-stream proteomics analysis also showed that in ischemic and nephrotoxic AKI, NGAL was the APP that increased when induced [4]. Ischemic AKI caused by cardiac surgery and kidney transplantation, nephrotoxic AKI caused by contrast agent, and septic AKI in critically ill patients all suggested that NGAL was a good biomarker for AKI early-stage diagnosis [6]. However, the function of NGAL expression in these events is unclear.

Previous studies indicated that as a lipocalin, NGAL could combine with and stabilize hydrophobic small molecular substances. Many human cancer cells can secrete NGAL. Yan’s group reported that NGAL could stabilize MMP-9 in neutrophilic granulocytes. NGAL covalently bind with MMP-9, and inhibited the degradation of MMP-9 and increased its activity. Also, MMP-9 promoted growth, infiltration and migration of carcinoma tissues by degrading the basement membrane and extracellular matrix, releasing vascular endothelial growth factors and promoting neonatal angiogenesis. Therefore, NGAL was considered to correlate with poor prognosis in cancer [14]. Morik and Schmidt reported that NGAL could generate an NGAL:ironophore, an iron complex which inhibited bacterial uptake of iron causing a bacteriostatic effect, and promoted kidney mesenchymal cells during the embryonic period to differentiate into proximal tubular cells. Furthermore, it also protected renal proximal tubular cells from hypoxic injury and death by up-regulating hemoxygenase [7, 15, 16]. Mishra’s group suggested that exogenous NGAL could protect renal proximal tubular cells, alleviate ischemia-reperfusion injury and inhibit apoptosis after injury [17]. Currently, NGAL during septic AKI is thought to have a protective effect but the mechanism is not understood; however, necrosis or apoptosis, which are consequences of irreversible injury [18–20] can be caused by numerous factors, and can co-occur. Whether necrosis or apoptosis predominates depends on the strength of the stimulating factor and the biological cellular characteristics [18, 21, 22]. Previous studies indicated that during AKI, apoptosis occurred with acute tubular necrosis (ATN). Lieberthal’s group has reported that cis-platinum to stimulate renal tubular epithelial cells induced apoptosis [23]. Furthermore, ATN has been verified in many ischemic AKI animal models, but this has not detected with a histologic examination of septic AKI, indicating that apoptosis may be the main cause of renal injury during septic AKI [10, 24].

Two mechanisms are said to cause apoptosis. First, a stressor (hypoxia or oxidative stress) stimulates mitochondria and decreases ATP generation and this is chiefly affected by intracellular changes in chemical information [25]. Another includes tumor necrosis factor (TNF) and TNF receptor (TNFR), which are mainly affected by extracellular stimulating information [26]. Caspases are proteases associated with apoptosis. Typically, they only exist in cells as cysteine proteases with low activity. When apoptosis was initiated by mitochondria or the TNF/TNFR pathway, caspase is activated by proteolysis to form apoptotic caspases 2, 3, 6, 7, 8, 9, and 10. A final apoptotic protease, caspase 3 can degrade intracellular proteins, creating an apoptotic body [27].

Guo’s group has suggested that apoptosis occurs in septic AKI induced by LPS and blood urea nitrogen is increased. About 3 h after LPS treatment, apoptotic cells were present in kidney tissues, and apoptosis was maintained for 48 h. In TNFR1 knockout mice, after the same treatment, apoptosis in renal tubular epithelial cells was significantly decreased, and kidney injury was alleviated. Therefore, in sepsis, LPS in kidney tissues mediated apoptosis by TNF/TNFR1, and apoptosis of epithelial cells was an important pathological pathway for kidney injury [28]. Also, LPS treatment increased blood urea nitrogen and renal caspase 3 activity increased. After a caspase 3 inhibitor was applied activity significantly decreased. Renal tubular epithelial apoptosis was reduced as was kidney injury, proving that apoptosis was an important injurious mechanism during septic AKI [29].

In our study, LPS was given as described in the Methods and AKI was established as evidenced by increased SCr and uNGAL. Histologically, injury was also confirmed by inflammatory cell infiltration in the renal interstitium, parenchymal injury in renal tubular epithelial cells in the renal cortex, cloudy swelling of the cytoplasm, the disappearance of the brush border, diminished lumen, and diminished or missing ligaments. Electron microscopy revealed apoptosis of renal tubular epithelial cells but not necrosis. Epithelial cells had contracted nuclear membranes and chromatin margination of apoptosis. In our study, septic AKI in rats did not cause necrosis of renal tubular epithelial cells but apoptosis did occur. Thus, apoptosis is an important pathological injury mechanism for septic AKI, and these data agree with that of Guo’s group [29]. Also, in injured renal tubular epithelial cells, NGAL and protein were significantly increased and correlated, indicating that early septic AKI involves NGAL up-regulation in renal tubular epithelial cells that is related to apoptosis. The meaning of this association, however, is unclear.

Using LPS to stimulate human proximal renal tubular epithelial cells and HK-2 cells, to observe the relationship between NGAL and caspase 3 at the genetic level, we found that 1–3 h after treatment, caspase 3 mRNA was significantly up-regulated and 1–6 h after LPS treatment, NGAL mRNA was up-regulated, indicating that apoptosis in HK-2 cells was initiated in a manner similar to rats with septic AKI and that NGAL and caspase 3 were linked.

Specific siRNA with homologous sequences can silence target gene expression via RNA interference (RNAi) [30–32]. To study the biological effect of NGAL in renal epithelial cells, specific NGAL siRNA was applied to silence the *NGAL* gene and LPS stimulation was applied (siRNA + LPS group) to observe changes in apoptosis. Caspase 3 mRNA was significantly increased compared to controls and the LPS-treated cells. Flow cytometry and TUNEL assay confirmed apoptotic cells in the siRNA + LPS was significantly greater than in other groups. Thus, when HK-2 cells were stimulated by LPS, intracellular NGAL was quickly synthesized and up-regulated by inhibiting caspase 3, which inhibited apoptosis to protect HK-2 cells from death. With septic AKI kidney injury arose from factors including LPS, hypoxia, oxidative stress and inflammatory factors. If caspase 3 production can be inhibited, injured cells may survive and permit renal healing and protect renal function. Inhibition of caspase 3 by NGAL in injured epithelial cells may be a promising target for treating septic AKI.

Our study is limited in that the data presented here represent only model of LPS-induced injury. More work is needed to explore underlying mechanisms of septic AKI, perhaps using organ-specific NGAL knock-out animals or NGAL antibodies in a septic AKI model induced by cecal ligation and puncture.

## Conclusions

This study provides evidence to suggest that NGAL is associated with caspase 3 in renal tubular cells with endotoxin-induced kidney injury, and may regulate its expression and inhibit apoptosis. These results need further confirmation in a septic AKI model.

## Abbreviations

AKI: Acute kidney injury
APP: Acute phase protein
ATN: Acute tubular necrosis
cDNA: Complementary DNA
FBS: Fetal bovine serum
H&E: Hematoxylin and eosin
HBSS: Hank’s Balanced Salt Solution
HPF: High-power field
IHS: Immunohistochemical staining
LPS: Lipopolysaccharide
MMP-9: Matrix metalloproteinase-9
NGAL: Neutrophil gelatinase-associated lipocalin
pNGAL: Plasma NGAL
RNAi: RNA interference
rt-qPCR: Real-time PCR
sAKI: LPS-induced AKI group
SCr: Serum creatinine
TEM: Transmission electron microscopy
TNF: Tumor necrosis factor
TNFR: TNF receptor
uNGAL: Urinary NGAL
